# NHE5 regulates growth factor signaling, integrin trafficking, and degradation in glioma cells

**DOI:** 10.1007/s10585-019-10001-6

**Published:** 2019-10-08

**Authors:** Toru Kurata, Vinotheni Rajendran, Steven Fan, Tetsuo Ohta, Masayuki Numata, Sachio Fushida

**Affiliations:** 1 Department of Gastroenterological Surgery, Kanazawa University Graduate School of Medical Science, 13-1 Takara-machi, Kanazawa, 920-8641 Japan; 2 Department of Biochemistry and Molecular Biology, University of British Columbia, Vancouver, BC V6T 1Z3 Canada

**Keywords:** β1-integrin, C6 glioma, EGFR, MET, NHE5

## Abstract

Na^+^/H^+^ exchanger 5 (NHE5) is enriched in neurons and cycles between recycling endosomes and plasma membranes and transports protons to the endosomal lumen as well as to the extracellular space. Although NHE5 expression is undetectable in normal astrocytes, C6 glioma cells express NHE5 at an elevated level. Using C6 cells as a model, here we demonstrate that NHE5 has an important role in tumor growth and tumor cell proliferation and invasion. Glioma xenografts originating from *NHE5*-knockdown cells exhibited significantly slower growth than those from *NHE1*-knockdown cells and control cells. Histological characterization of the migration front of *NHE5*-knockdown tumors revealed a less invasive and less proliferative appearance than *NHE1*-knockdown and control tumors. *NHE5*-knockdown but not *NHE1*-knockdown led to downregulation of fetal bovine serum (FBS)-induced MET and EGFR signaling. Moreover, depletion of NHE5 but not NHE1 reduced the ability of cells to spread on collagen. We found that NHE5 depletion greatly abrogated endocytic recycling and the protein stability of β1-integrin, which in part accounted for the defective cell adhesion, spreading, and invasion of *NHE5*-knockdown cells.

## Background

Acidic extracellular pH is a hallmark of the tumor microenvironment, where proton-pumping activity in malignant tumor cells has a critical role in establishing extracellular acidity by exporting protons produced by tumor metabolism [1]. Na^+^/H^+^ exchanger NHE1 is one of the most extensively studied pH-regulating ion transporters [2, 3]. Since the discovery of NHE1 as a growth factor-activatable electroneutral antiporter that secretes protons from the cytosol to the extracellular space [4], 12 additional NHE genes have been identified in the human genome [5, 6]. Among the different NHE isoforms, NHE1 has received the most attention as a potential target for anti-cancer therapy. Although previous studies showed the importance of NHE1 in cell proliferation [7–10], little is known about the involvement of other NHE isoforms that may be expressed in malignant tumor cells.

NHE5 was originally identified as NHE whose mRNA [11, 12] and protein expression [13] were detected in the brain, particularly in neuron-enriched brain regions. More recently, NHE5 protein was shown to be abundantly expressed in C6 glioma cells while NHE5 being undetectable in normal astrocytes [14]. NHE5 cycles between recycling endosomes and the plasma membrane [15], and acidifies the lumen of recycling endosomes [16], thereby playing a critical role in regulating the cell-surface availability of the high-affinity nerve growth factor receptor TrkA in PC12 pheochromocytoma cells of adrenal gland origin [17] and the hepatocyte growth factor/scatter factor (HGF) receptor MET in C6 glioma cells [14]. In the current study, we have investigated the involvement of NHE5 and NHE1 in glioma cell signaling, proliferation, and tumor growth through characterization of C6 glioma-based knockdown cell lines.

## Materials and methods

### Molecular biology

Plasmid-based short hairpin RNA (shRNA) constructs against *NHE1*, *NHE5*, and scrambled control (N5shA, N5shB, and N5shC, respectively), and C6 cell lines stably expressing shRNA were described previously [14]. Unless otherwise stated, C6 cells expressing N5shA were used as a representative *NHE5*-knockdown cell line. Human integrin β1 (*hITGB1*) cDNA was amplified from pMD-*ITGB1* (#HG10587-M; Sino Biological, Beijing, China) using the following primers: 5′-CCTCGAAAGGCCTCTGAGGCCATGAATTTACAACCAATTTTCTGG-3′ and 5′-GGAAGCTTGGCCTGACAGGCCTCATTTTCCCTCATACTTCGG-3′. The PCR amplicon was then ligated into a mammalian expression vector pSBtet-Pur [18] (Addgene, Cambridge, MA, USA). Modified site-directed mutagenesis [19, 20] was employed to insert an HA-tag after the 24th amino acid residue of *hITGB1* using the following primers: 5′-CCATATGACGTGCCCGACTACGCCGGAGAAAATAGATGTTTAAAAGCAAATGCC-3′ (sense) and 5′-GGCGTAGTCGGGCACGTCATATGGGTATTCATCTGTTTGAGCAAACAC-3′ (antisense).

### Semi-quantitative determination of MET and EGFR signaling

Serum-starved cells were stimulated by adding FBS to culture media to a final concentration of 10%. Cells were then washed with PBS and lysed in radioimmunoprecipitation assay (RIPA) buffer (50 mM Tris–HCl, 50 mM NaCl, 0.1% SDS, 0.5% sodium deoxycholate, and 1% nonidet P-40; pH 7.2) supplemented with protease inhibitor (11697498001; Sigma Aldrich, St. Louis, MO) and phosphatase inhibitor (4906845001, Sigma Aldrich). Debris-cleared lysates were mixed with SDS sample buffer (125 mM Tris–HCl, 4% SDS, 20% glycerol, 0.004% bromophenol blue, and 10% dithiothreitol; pH 6.8) and denatured at 65 °C for 20 min. Equal amounts of proteins were subjected to SDS-PAGE, followed by western blotting. Antibodies against tyrosine-and/or threonine-phosphorylated EGFR (pY1068, 3777; Cell Signalling Technology, Danvers, MA), MET (pY1234/Y1235, 44-888G; Thermo Fisher, Waltham, MA), ERK1/2 (pT202/Y204, 9101; Cell Signalling Technology), and AKT (pT308, 1308, Cell Signalling Technology) were used to detect the phosphorylation status of these signaling molecules. Blots were reprobed with antibodies against EGFR (sc-03; Santa Cruz Biotechnology, Dallas, TX), MET (3127; Cell Signalling Technology), AKT (9272, Cell Signalling Technology), ERK1/2 (9102; Cell Signalling Technology), NHE1 (611774; BD Biosciences, San Jose, CA), NHE5 (GenScript, Piscataway, NJ) [13, 16], and Na^+^/K^+^-ATPase (NKA, α5; Developmental Studies Hybridoma Bank, Iowa City, IA).

### Spheroid growth assays

C6 spheroid cultures were generated according to the liquid overlay method, as previously described [21]. Ninety-six-well flat-bottomed plates were coated with a 1:1 mixture of dissolved 1% agarose and pre-warmed DMEM. After the agarose mixture had solidified, 500 cells/100 μL culture media were added to each well and the plates were centrifuged at 1500×*g* for 10 min, followed by incubation at 37 °C in 5% CO_2_. Images of spheroids were captured with a light microscope. Spheroid growth was assessed by acid phosphatase assay (APH), as previously described [22].

### Determination of cell attachment

Auguiar et al. showed that C6 cells exhibit stronger attachment and greater migration on Collagen IV than on other ECM proteins (laminin and fibronectin) [23]. This study has provided us with the rational in using collagen IV as ECM. Two-thousand cells were seeded onto collagen IV (col IV; 0.06 μg/mL)- or polyethyleneimine (PEI; 25 mM)-coated 96-well plates. After the cells had been allowed to spread for various time periods, non-adherent cells were gently washed away with PBS and cell attachment was terminated by fixation in 3% PFA supplemented with 500 ng/mL Hoechst-33342 dye (Sigma Aldrich) for 15 min at room temperature. Images were captured and analyzed by a Cellomics Arrayscan VTI high content screening system (Thermo Fischer Scientific) according to the protocol provided by the manufacturer.

### Measurement of cell spreading areas

Cells spread onto PEI- or col IV-coated coverslips were fixed, permeabilized, and incubated with Alexa-Fluor-488-conjugated Alexa 488-phalloidin and DRAQ5 to visualize the actin cytoskeleton and nuclei, respectively. Images were captured with a Leica TCS-SP8 laser scanning confocal microscope and analyzed by Fuji Is Just ImageJ (Fuji) software. The brightness and contrast of the images were auto-adjusted, followed by determination of the auto-threshold of the signal for these images to generate binary images. The area of the cells was measured using the ‘measure’ plugin of the software.

### Cell surface biotinylation and endocytosis assays

Cell surface biotinylation and endocytosis were assessed as described previously [14] with some modifications. Subconfluent cells seeded onto fibronectin-coated plates were labeled with 0.3 mg/mL EZ-Link™Sulfo-NHS-SS-Biotin (Thermo Fisher Scientific) for 35 min at 4 °C, followed by quenching by PBS supplemented with 20 mM glycine. Cells were then incubated in conditioned growth medium at 37 °C for 0 or 10 min to allow for internalization of the cell surface proteins. Biotin was removed from the non-internalized cell surface proteins using ice-cold membrane-impermeable cleavage buffer containing 50 mM glutathione. Some samples were not subjected to the cleavage step (‘non-cleaved proteins’) to determine intrinsic degradation during incubation at 37 °C. Next, 30 µg of non-cleaved proteins and 160 μg cleaved proteins were incubated with Pierce™NeutrAvidin™-conjugated agarose beads at 4 °C. The eluted protein samples as well as 6 μg of the total protein were analyzed by western blotting. Densitometry analysis was performed using Image J software. The following equation was used to determine the internalization percentage: [10′(+) − 0′(+)]/10′(−), where 10′(+) and 0′(+) represent the densitometric signal of biotinylated proteins that were treated with cleavage buffer after 10 min and 0 min of post-biotinylation incubations, respectively. 10′(−) represents the biotinylated proteins (not treated with cleavage buffer) after 10 min of post-biotinylation incubation, which includes both internalized and non-internalized populations.

### Degradation assays

Cells were seeded overnight and cell surface proteins were biotinylated as described above. After quenching, the cells were incubated at 37 °C in a humidified atmosphere with 5% CO_2_ for the indicated times, followed by rinsing twice with 1 × PBS (pH 7.4) and lysing with ice-cold RIPA buffer supplemented with proteinase inhibitor cocktails. Approximately 50–60 μg of total protein was incubated with Pierce NeutrAvidin™ agarose beads overnight at 4 °C with rotation. The beads were washed three times with ice-cold 1% NP-40 in PBS (pH 7.4) and the absorbed protein was eluted with 2 × Laemmli sample buffer. The eluted protein and approximately 6 μg of the total protein were immunoblotted for in integrin β1. Densitometry analysis was performed using Image J software. The relative pool of remaining biotinylated integrin β1 at the indicated time was obtained by normalizing the value to that of time 0 in the respective cell lines.

### Recycling assays

Recycling assays were performed as described previously [14] with some modifications. After cell surface biotinylation, chase incubation, and cleavage, cells were subjected to a second round of incubation at 37 °C for 10 min to allow the internalized biotin-labeled proteins to be recycled back to the cell surface. Treatment of cells with cleavage buffer led to the removal of biotin from proteins that had been recycled to the cell surface. The cells were then rinsed several times with ice-cold PBS-CM (PBS containing 0.1 mM of CaCl_2_ and 1 mM of MgCl_2_, pH 8.0) and lysed. To determine the intrinsic degradation, some samples were not subjected to the second post-incubation cleavage. Approximately 450 μg of protein was incubated with Pierce NeutrAvidin™-conjugated agarose beads. The eluted protein samples as well as ~ 6 μg of the total protein that was not subjected to the NeutrAvidin™ agarose bead-pulldown were analyzed by immunoblotting. Densitometric analysis was performed using Image J software. The following equation was used to assess the ratio of recycled proteins after 10 min (10′) of second incubation at 37 °C: 1− (10′(+)/10′(−)), in which 10′(+) is the densitometric signal of sample treated with second post-incubation cleavage while 10′(−) is the corresponding sample without second post-incubation cleavage.

### Antibody feeding assays and colocalization analysis

*HA*-*hITGB1* expression was induced by 200 ng/mL doxycycline and labeled by adding anti-HA antibody to the bathing solution of cells. After quick washing, cells were incubated in conditioned complete medium at 37 °C for 10 min. Intracellular localization of *HA*-*hITGB1* and *EEA1* was determined by immunofluorescence microscopy. Nuclei were visualized by DRAQ5™ (#62254; Thermo Fisher). The images were acquired using Leica TCS-SP8 laser scanning confocal microscope equipped with × 63 oil immersion objectives (NA 1.40) with a zoom factor of 2.80, Diode/Argon/HeNe lasers, and HyDTM hybrid detectors (Wetzlar, Germany). The pixel sizes of the images were 0.129 μm. Confocal microscopic images were analyzed using Just Another Colocalization Plugin (JACoP) in Fiji Is Just ImageJ (Fiji) software to obtain the Pearson’s correlation coefficient between the signals.

### Invasion assays

The invasive ability of each cell line was assessed using BD BioCoatMatrigel Invasion Chambers for 24-well plates (BD Bioscience, Franklin Lakes, NJ, USA), according to the manufacturer’s instructions. In brief, Matrigel on the polycarbonate membrane with 8 μm pore was rehydrated using 750 μL of serum-free medium, after which 750 μL of fresh medium containing 10% FBS as chemoattractant was added to the lower chamber. Next, 0.5 mL of each cell line (1 × 10^5^ cells/mL) in serum-free medium was seeded into the upper chamber. After 24 h, cells in the upper chamber were removed, and those that had invaded through the Matrigel were fixed in 100% methanol and stained with hematoxylin. Using three membranes per group, invading cells were counted in several fields under a microscope with a 10 × objective. The following equation was used to calculate the proportion of invading cells: % invasion = (mean number of cells invading through Matrigel/mean number of cells migrating through Matrigel-uncoated polycarbonate membrane) × 100.

### Mouse subcutaneous xenograft models

Animals were treated in accordance with the Fundamental Guidelines for Proper Conduct of Animal Experiment and Related Activities in Academic Research Institutions, under the jurisdiction of the Ministry of Education, Culture, Sports, Science and Technology of Japan. All animal experiments were approved by the Committee on Animal Experimentation of Kanazawa University. Female BALB/c-nu/nu mice (Charles River Laboratories, Inc., Yokohama, Japan) of 4 weeks of age were maintained in a sterile environment. *NHE1*-knockdown, *NHE5*-knockdown and control cells were cultured in DMEM with 10% FBS and appropriate selection antibiotics (G418 and hygromycin) to subconfluency, and 5 × 10^6^ cells in 100 μL of DMEM without FBS were subcutaneously injected into the dorsal side of each mouse. Animals were carefully monitored, and tumor volumes were measured on days 4, 6, 8, and 10. The tumor volume (V) was calculated according to the formula V = AB^2^/2, where A is the length of the major axis, and B that of the minor axis. On day 10, mice were killed and tumor specimens were collected for histological and immunohistochemical examination.

### Histology and immunohistochemistry

Tumor specimens were fixed in 10% neutral buffered formalin and embedded in paraffin. Sections were stained with hematoxylin and eosin (HE) for assessment of invasiveness into the surrounding tissue. Deparaffinized sections were pretreated by autoclaving in 10% citric acid buffer (pH 8.0) at 120 °C for 15 min. Following treatment with protein block serum (DakoCytomation) for 10 min and 2% skim milk for 30 min to block non-specific reactions, sections were incubated with the primary antibody at 4 °C overnight. EnVision polymer solution (HRP; DakoCytomation) was then applied for 1 h. Proteins were visualized with 0.02% 3, 3′-diaminobenzidinetetrahydrochloride solution. Sections were then lightly counterstained with hematoxylin.

### Determination of Ki-67 scores

Anti-Ki-67 antibodies (M7248, diluted 1:25; DakoCytomation, Kyoto, Japan) were used for immunohistochemical assessments. Slides were scanned and higher density-stained areas were located using an Olympus microscope. Three independent authors (TK, SF, and MN) manually counted at least 2000 tumor cells in three randomly chosen microscopic fields for calculating the average percentage of positive tumor cells.

## Results

### MET and EGFR signaling are impaired in *NHE5*-knockdown glioma cells

**Fig. 1:**
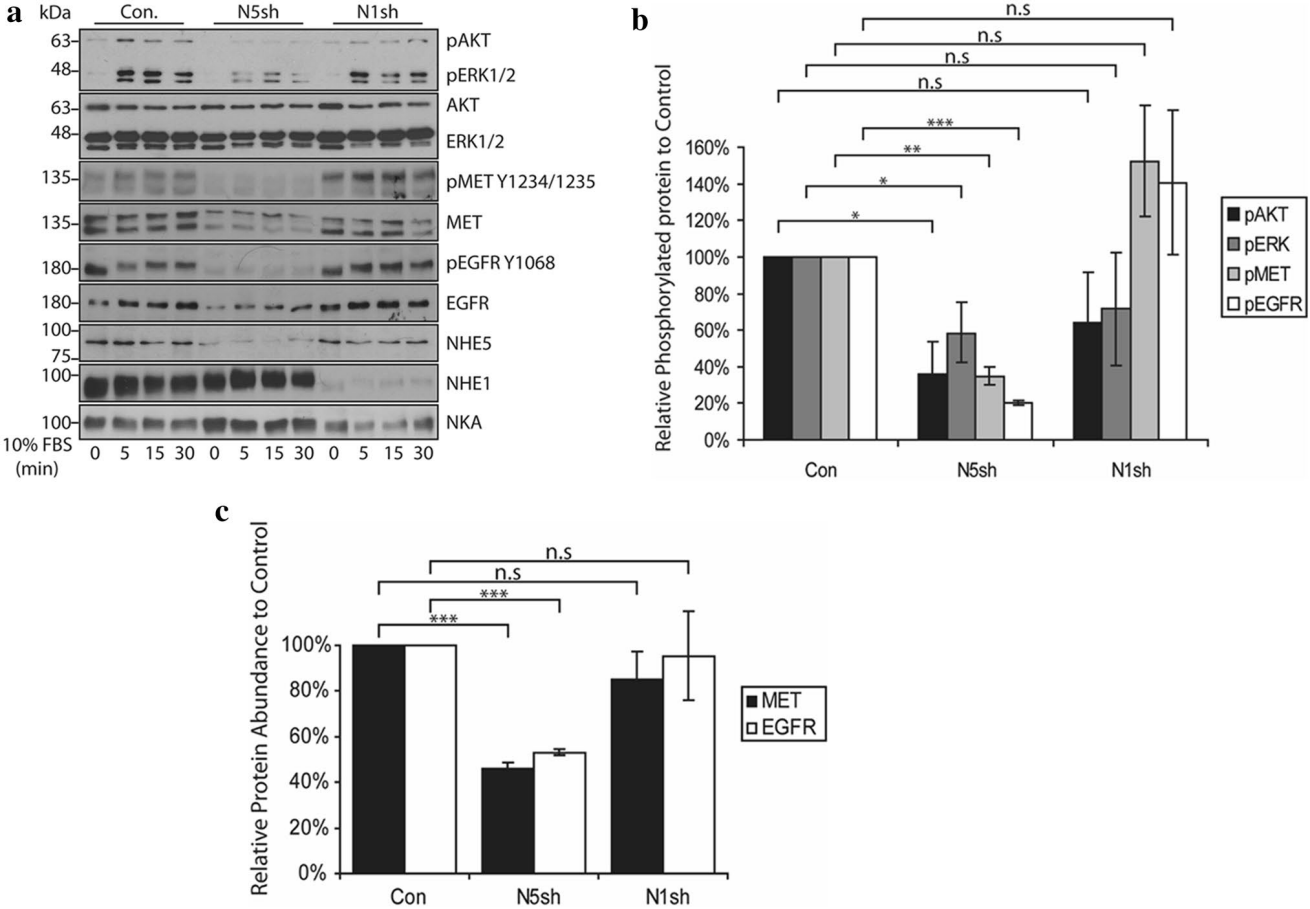
NHE5 depletion dramatically downregulates phosphorylation of MET and EGFR and attenuates PI3 K and MAPK signaling. **a** Serum-starved C6 cells stably expressing shRNA plasmid against *NHE5* (N5sh), *NHE1* (N1sh), or scrambled sequence (Con) were stimulated with culture medium containing 10% FBS for 0, 5, 15, or 30 min and analyzed by western blotting. Activation of AKT, ERK1/2, MET, and EGFR and was assessed by antibodies that specifically recognize the phosphorylated forms of the respective proteins. **b** Densitometric analysis of phosphorylated AKT, ERK1/2, MET, and EGFR at 15 min post FBS stimulation is shown. Band intensities of phosphorylated proteins from *NHE5*-KD (N5sh), *NHE1*-KD (N1sh), or control (Con) cell lysate were normalized to that of control. Mean ± SD of three experiments are shown. *P* Values were obtained from Student’s *t* test. *p < 0.05, **p < 0.01, ***p < 0.001, *n.s* not significant). **c** Densitometric analysis of total MET and EGFR protein from *NHE5*-KD (N5sh), *NHE1*-KD (N1sh), or control (Con) cells is shown. Band intensities of total MET or EGFR from respective cell lysates were normalized to that of control cell lysate. Mean ± SD of three experiments are shown. *P* Values were obtained from Student’s *t* test. *p < 0.05, **p < 0.01, ***p < 0.001, *n.s* not significant)

FBS stimulation of C6 glioma cells led to phosphorylation of MET, EGFR, AKT, and ERK1/2 (Fig. 1). When serum-starved *NHE5*-knockdown cells were stimulated with FBS, phosphorylation of MET and EGFR were barely detectable, and phosphorylation of AKT and ERK1/2 were also greatly diminished. A slight but a significant decrease in the total protein levels of MET and EGFR was also detectable in NHE5-depleted cells. *NHE1* knockdown resulted in a slight reduction in the phosphorylation of AKT and ERK1/2, whereas there was no effect on MET and EGFR phosphorylation.

### Proliferation in 3D spheroid cultures is impaired in *NHE5*-knockdown glioma cells

**Fig. 2:**
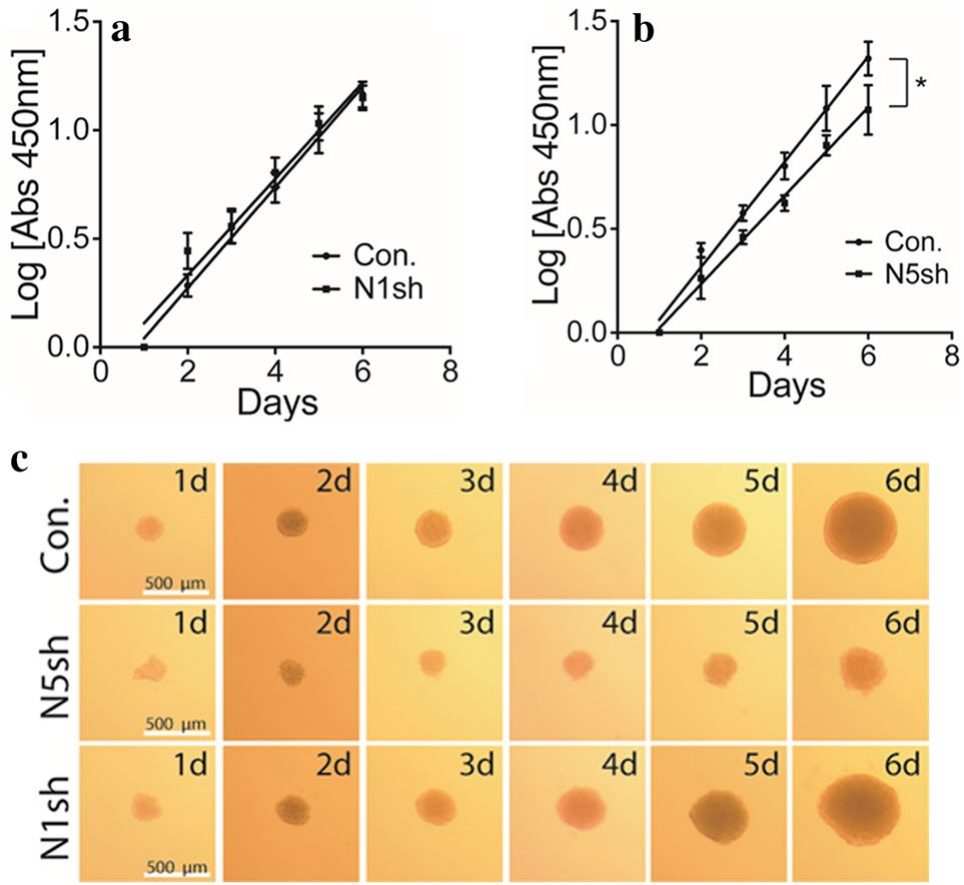
Depletion of NHE5 reduces the growth of spheroid cultures. **a**, **b** Log-transformed growth curves of spheroid cultures of C6 cells stably expressing shRNA against *NHE1* (N1sh), *NHE5* (N5sh), and scrambled sequence (Con) over the indicated times. The growth of spheroid cultures was assessed by APH assay. Measured Absorbance at 450 nm of indicated time for each cell line were converted to log scale and plotted on Graphpad. Best-fitted curve of NHE5- or NHE1-knockdown cells were compared to control cells by linear regression analysis, where slopes and intercepts of the resulted curves were compared. Graphs represent mean ± SD of quadruplicate samples (*P < 0.05). **c** Representative microscopic images of spheroid cultures (bars = 500 μm)

Spheroid cultures reflect the 3D cellular context and can serve as an excellent model to characterize the cellular behavior of tumor tissues [22]. Proliferation of *NHE5*-knockdown spheroids was significantly slower than that of the control cells, whereas proliferation was not affected by *NHE1*-knockdown (Fig. 2a–c).

### Spreading on collagen is impaired in *NHE5*-knockdown glioma cells

**Fig. 3:**
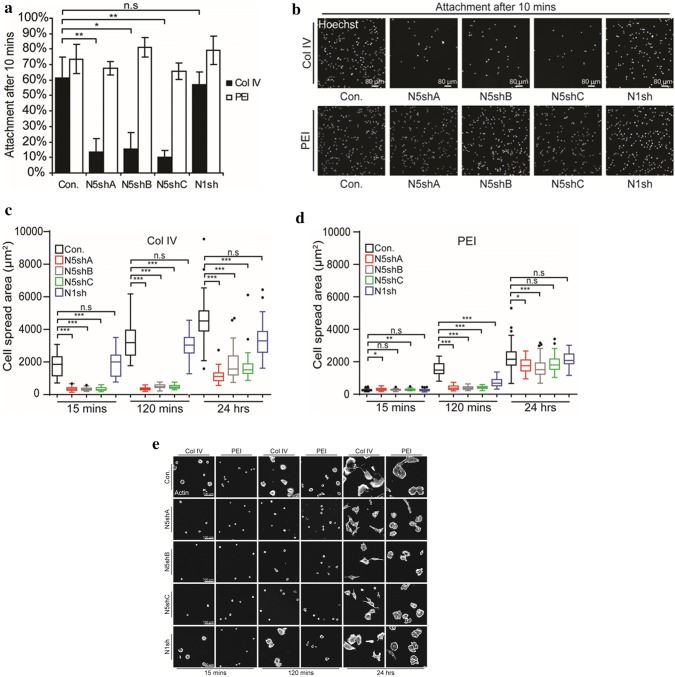
*NHE5* knockdown impedes cell attachment and spreading. **a** C6 cells stably expressing shRNA against *NHE5* (N5shA, B, C), *NHE1* (N1sh), or scrambled control (Con) were subjected to cell attachment assays on collagen IV (col IV)- or polyethyleneimine (PEI)-coated surfaces. Data are presented as percentages of cells attached to different substrates after 10 min of spreading. Means and standard deviations are plotted (*P < 0.05; **P < 0.01; *n.s* not significant; n = 3) bar = 80 μm. **b** Representative images of control, *NHE5*-knockdown, and *NHE1*-knockdown cells spread onto a col IV- or PEI-coated plate for 10 min. **c**, **d** The spread area of randomly selected cells was analyzed (n > 40) and the distribution is shown as box-and-whisker plots with the ends of the whiskers set at 1.5 × interquartile range (IQR) above the third quartile and 1.5 × IQR below the first quartile. Kruskal–Wallis tests with Dunn’s tests with Bonferroni correction for pair-wise comparison were performed. (*P < 0.05; **P < 0.01; ***P < 0.001; n.s = not significant). **e** Representative confocal images of control, *NHE5*-, or *NHE1*-knockdown cells on col IV- or PEI-coated surfaces after 15 min. Bar = 100 μm

Cell spreading and adhesion to extracellular matrices are important for cell survival, cell migration, and tissue remodeling [24]. To examine the capability of cells to adhere to different matrices, equal numbers of *NHE5*-knockdown cells, *NHE1*-knockdown cells, and control cells were seeded onto glass coverslips coated with either PEI (non-integrin matrix) or col IV (integrin matrix). The number of *NHE5*-knockdown cells (N5shA, N5shB, and N5shC) attached to col IV-coated coverslips after 10 min of seeding was significantly smaller than that of scrambled control cells (Con) and *NHE1*-knockdown cells (N1sh) (Fig. 3a, b). There was no apparent difference in the number of cells attached to PEI-coated coverslips among the different cell lines. We next quantitatively determined the cell spreading area of *NHE5*-knockdown cells, *NHE1*-knockdown cells, and control cells using confocal microscopy. The same number of cells was seeded onto col IV- or PEI-coated coverslips and incubated at 37 °C for different time durations. Cells were then fixed, and the area to which cells were attached was calculated. Spreading areas of the three *NHE5*-knockdown cell groups expressing different shRNAs (N5shA, N5shB, and N5shC) were notably smaller than those of *NHE1*-knockdown cells and control cells when cells were spread over col IV-coated coverslips (Fig. 3c, e). In contrast, when cells were spread over PEI-coated coverslips, there was no apparent difference in the areas spread by the different cell lines (Fig. 3d, e).

### *NHE5* knockdown diminishes endocytic recycling of integrin β1

**Fig. 4:**
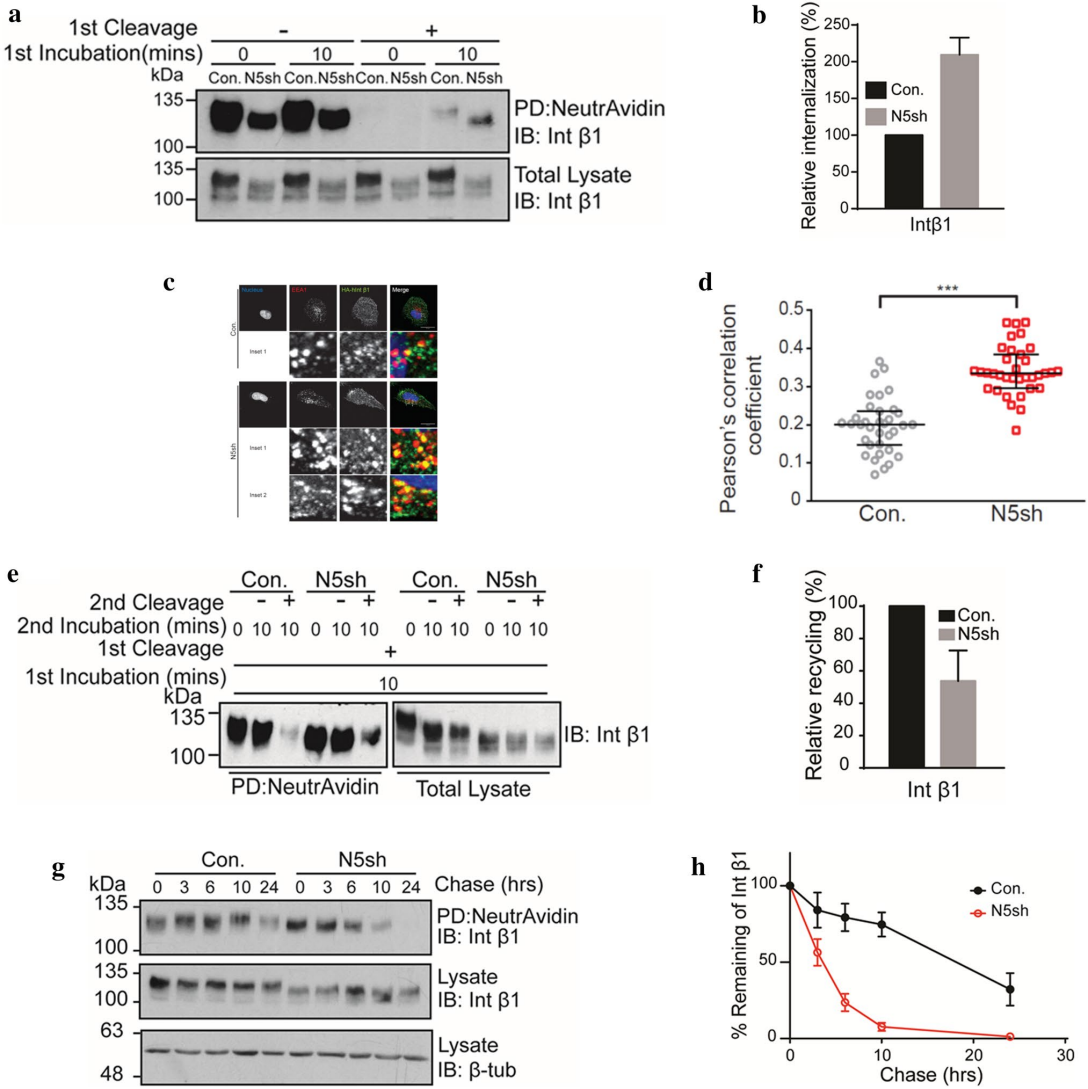
NHE5 regulates intracellular trafficking of integrin β1. **a** Representative data of cell surface expression and internalization of integrin β1. **b** Relative internalization of integrin β1 in *NHE5*-knockdown cells and control cells. Means and standard deviations are shown (n = 3). **c** Representative confocal images of the antibody feeding assays. Localization of internalized HA-tagged integrin β1 (HA-hITGB1, green) in *NHE5*-knockdown cells and control cells was analyzed by immunofluorescence microscopy. Early endosomes and nuclei are marked by anti-EEA1 antibody (red) and Hoechst dye (blue), respectively. Bars = 20 μm. The zoomed insets (white squares) that show the colocalization between EEA1 and HA-hITGB1. **d** Scatter plot of Pearson’s correlation coefficient of colocalization between HA-hITGB1 and EEA1 in control and *NHE5*-knockdown cells. The median and interquartile range are shown (***P < 0.001; Mann–Whitney test; n = 2). **e** Representative result of integrin β1 recycling assays. Recycling of cell surface biotinylated and internalized proteins was determined. Following the ‘second cleavage’, only non-recycled integrin β1 that was protected from cleavage was detected. **f** Relative recycling was calculated by normalizing the recycled pool of *NHE5*-knockdown cells to that of control cells. Means and standard deviations are shown (n = 3). **g** Representative result of integrin β1 degradation assay. **h** Relative degradation of integrin β1 in control or *NHE5*-knockdown cells plotted over time. Mean ± SD from three independent experiments are shown

Integrins are heterodimeric transmembrane receptors consisting of α and β subunits and facilitate cell attachment to extracellular matrices such as collagens [25]. The β1 subunit is abundantly expressed in C6 cells and has an important role in cell adhesion and spreading on collagen [26]. We next investigated the possible role of NHE5 in trafficking and its effect on the stability of integrin β1. The abundance of integrin β1 in the plasma membrane of *NHE5*-knockdown cells was significantly lower than that in control cells (Fig. 4a). To examine the effect of *NHE5* knockdown on endocytic trafficking of integrin β1, we performed cell surface biotinylation and endocytosis assays. In these experiments, the surface of the cells was biotinylated followed by chase incubation to facilitate endocytosis of the biotin-labeled membrane proteins. Cells were then treated with a membrane-impermeable reducing agent that selectively removes biotin from non-internalized proteins present on the plasma membrane. Treatment of *NHE5*-knockdown cells and control cells with the reducing agent resulted in almost complete disappearance of biotinylated integrin β1 (Fig. 4a). When cells were subjected to post-biotinylation chase incubation, the internalized integrin β1 population that was not accessible to the cleavage buffer became detectable by western blotting. Interestingly, the intensity of integrin β1 was substantially stronger in *NHE5*-knockdown cells than in the control cells, suggesting that there is a larger population of internalized integrin β1 in *NHE5*-knockdown cells (Fig. 4a, b). To further investigate this possibility, *NHE5*-knockdown cells and C6 cells stably expressing N-terminally HA-tagged human integrin β1 (*HA*-*hITGB1*) driven by the tetracycline-inducible promoter were established. Based on the resolved 3D structure, we designed the position of the HA-tag so that it was exposed to the extracellular space of integrin β1 [27]. *NHE5*-knockdown cells and control cells stably expressing inducible *HA*-*hITGB1* were cultured overnight at 37 °C in complete medium containing 200 ng/mL of doxycycline, and HA-hITGB1 protein residing in the plasma membrane was labeled *in cell* at 4 °C by adding anti-HA antibody to the bathing solution. After extensive washing and quenching, cells were subjected to incubation at 37 °C to render cell surface receptors subject to endocytosis. The cells were then fixed and the intracellular localization of HA-hITGB1 was determined by immunofluorescence microscopy. Endocytosed integrin β1 exhibited a higher degree of colocalization with an early endosomal marker EEA1 in *NHE5*-knockdown cells than in control cells (Fig. 4c, d). As decreased endocytic recycling may increase the endosomal population of receptor proteins, we next examined endocytic recycling. Cell surface biotinylated proteins were internalized, and the non-internalized population was removed by treating cells with cleavage buffer containing a membrane-impermeable reducing agent. The cells were then subjected to a second round of chase incubation to facilitate recycling from endosomes to the plasma membrane, followed by the treatment with cleavage buffer (‘second cleavage’; Fig. 4e) to eliminate biotin from proteins that have returned to the plasma membrane. Finally, biotinylated proteins were affinity-purified and integrin β1 was detected by western blotting. The signal intensity of biotinylated integrin β1 that represents the non-recycled population was higher in *NHE5*-knockdown cells than in the control cells, indicating that integrin β1 recycling is impaired in *NHE5*-knockdown cells (Fig. 4e, f).

### *NHE5*-knockdown accelerates degradation of integrin β1

Internalized receptors can be either recycled back to the plasma membrane or targeted to lysosomes for degradation. It was previously shown that degradation of integrin β1 is accelerated when its recycling is perturbed [28], which led us to hypothesize that NHE5 influences degradation of integrin β1. Integrin β1 was pulse-labeled by cell surface biotinylation and the degradation rate was determined after chase incubation for different durations. The half-life of integrin β1 in control cells was approximately 18 h whereas that of *NHE5*-knockdown cells was approximately 4 h (Fig. 4g, h).

### NHE5 is required for cell invasion through Matrigel

**Fig. 5:**
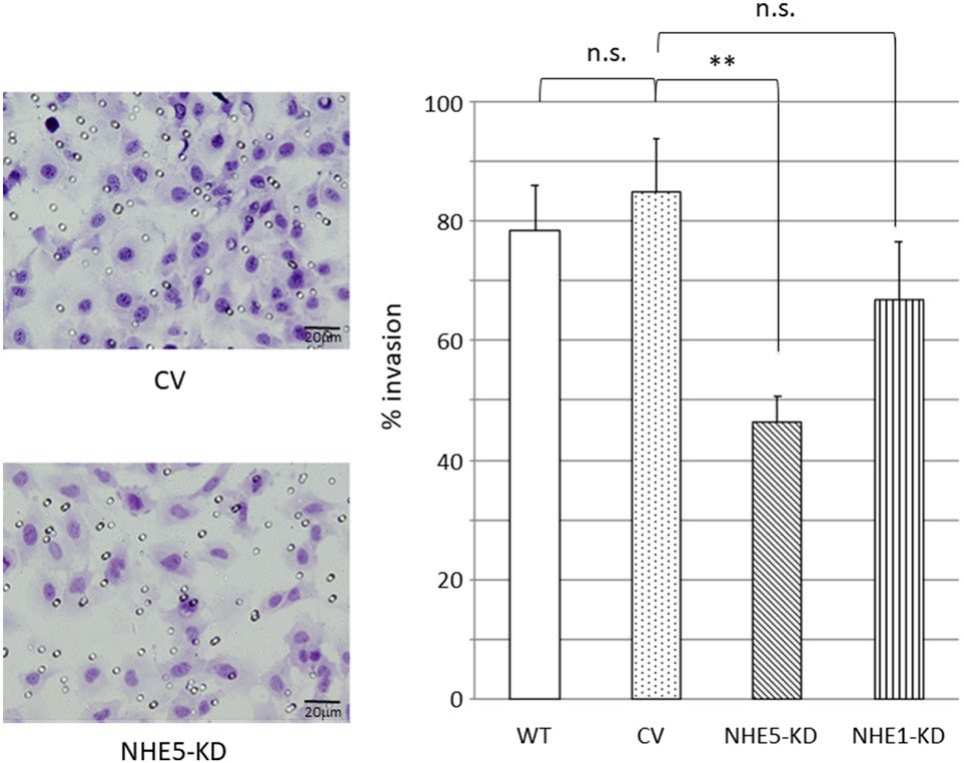
Invasion assays. (left) Representative images of invaded C6 cells expressing control vector (upper; CV) and *NHE5*-knockdown cells (lower; NHE5-KD). (right) Invaded cells in each clone were counted. (**P < 0.01; *n.s.* not significant). WT, wild type NHE; CV, control vector transfection; NHE5-KD, *NHE5*-knockdown; NHE1-KD, *NHE1*-knockdown

Endocytosis and recycling of integrins are increasingly recognized as crucial regulatory events of cell migration and invasion by regulating the balance between cell-surface availability and degradation of membrane proteins, which led us to examine invasion capability of *NHE5*-knockdown cells [29]. Invasion capability across Matrigel was substantially slower in *NHE5*-knockdown cells compared with *NHE1*-knockdown cells and control cells (Fig. 5). Because BD Matrigel contained some growth factors, such as TGF-beta, EGF and IGF etc., our invasion assay cannot be entirely distinguished from chemotaxis assay. However, these results suggest that NHE5 plays an important role in invasion through matrices.

### *NHE5*-knockdown reduces the rate of xenograft tumor growth

**Fig. 6:**
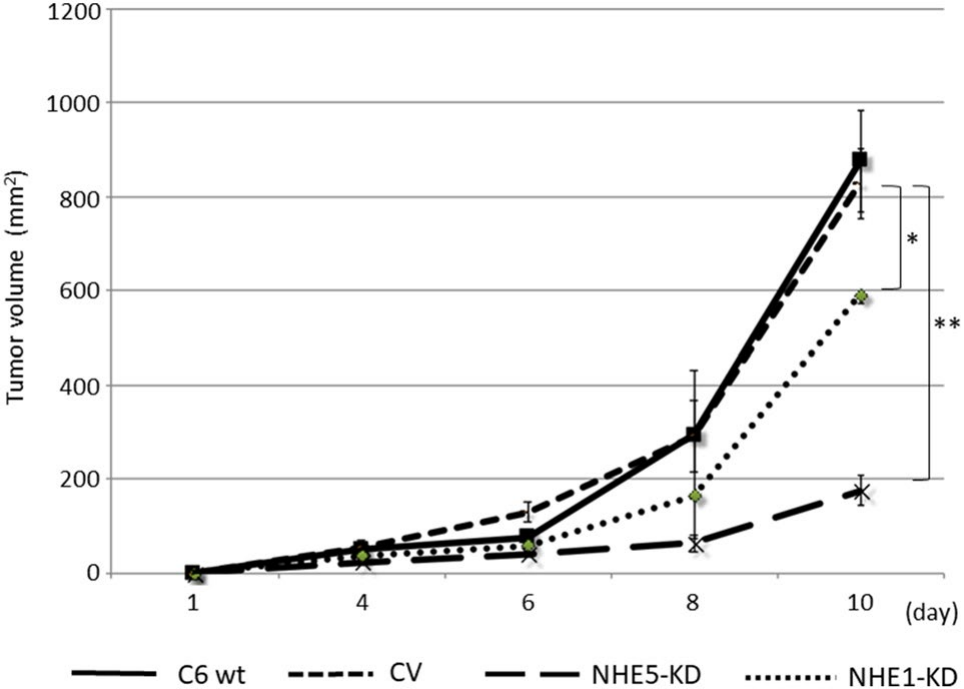
Tumors derived from NHE knockdown cells were significantly smaller than those from control vector. (*P < 0.05; **P < 0.01)

**Fig. 7:**
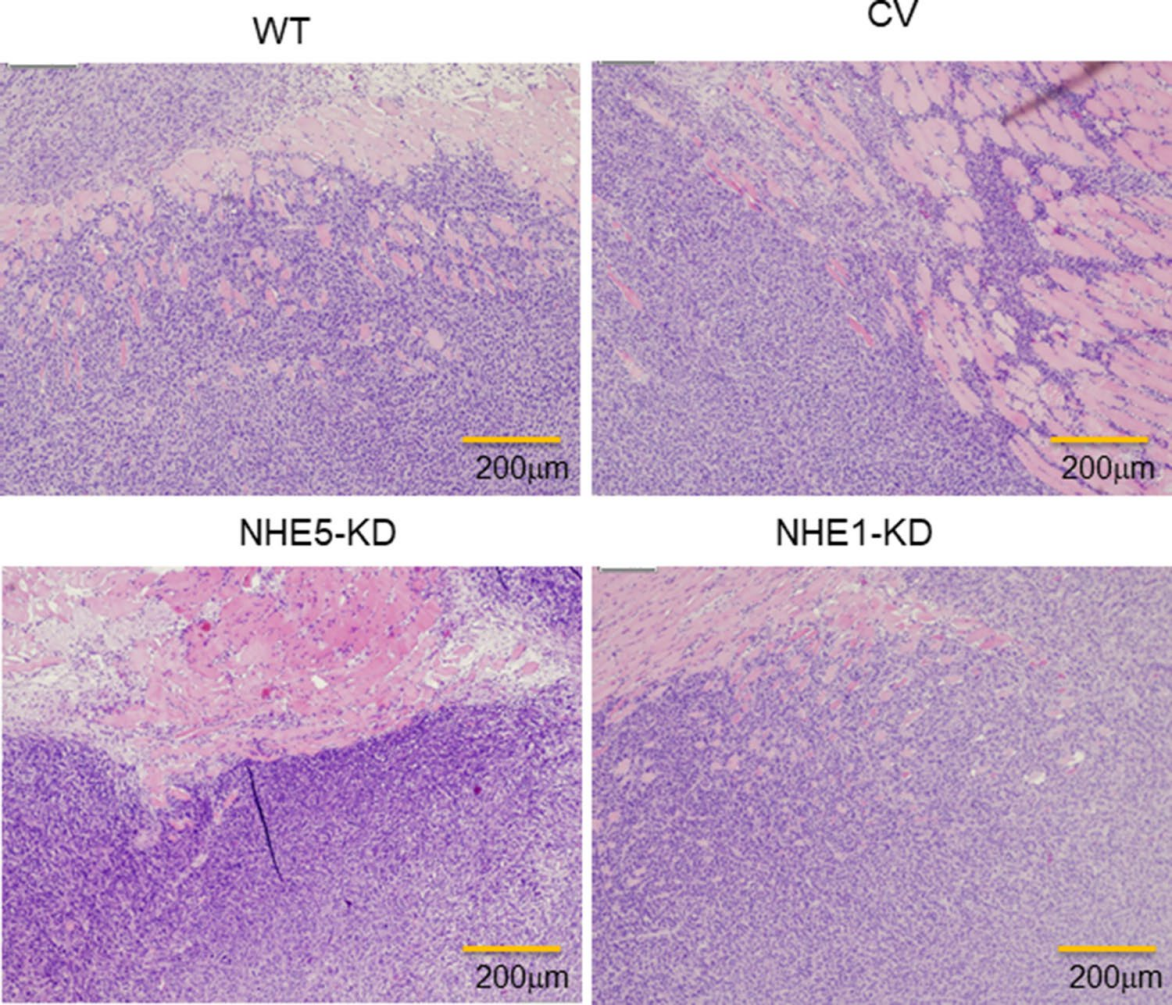
Histological examination by hematoxylin and eosin staining. WT, wild type; CV, control vector; NHE5-KD, *NHE5*-knockdown; NHE1-KD, *NHE1*-knockdown. (Magnification, × 40)

**Fig. 8:**
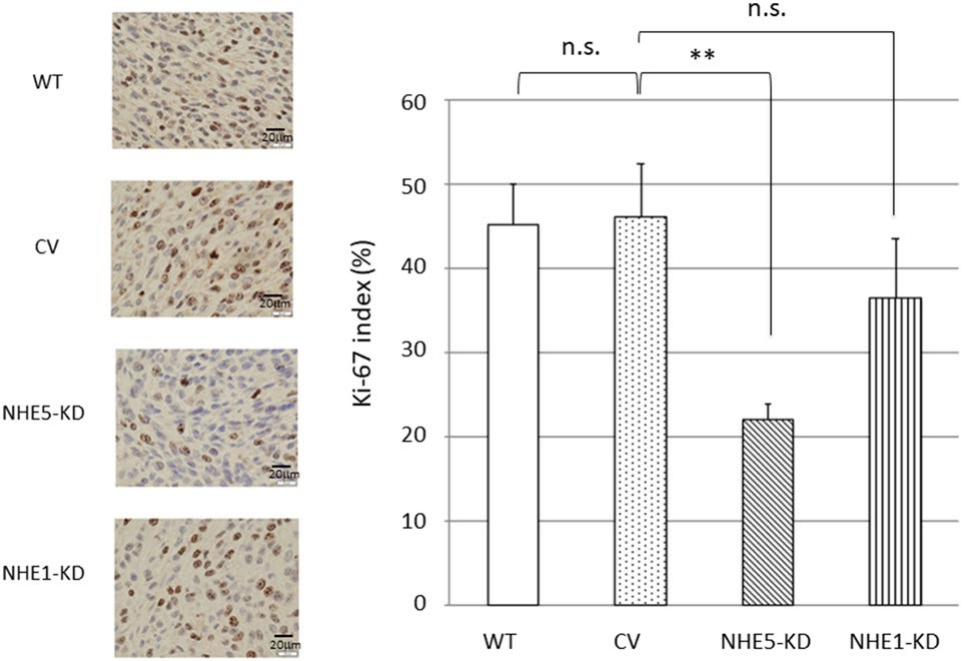
Ki-67 labeling index. (left) Representative photomicrograph of Ki-67-labeled cells in each tumor. (right) Ki-67 labeling index of each tumor. (**P < 0.01; *n.s.* not significant). WT, wild type NHE; CV, control vector transfection: NHE5-KD, *NHE5*-knockdown; NHE1-KD, *NHE1*-knockdown

The abovementioned results have indicated the possible involvement of NHE5 in tumor growth and progression. We next examined the growth of tumors derived from *NHE5*-knockdown cells, *NHE1*-knockdown cells, and control cells in vivo. The volume of *NHE5*-knockdown (NHE5-KD) tumors was markedly smaller than that of tumors originating from parental C6 cells (C6 wt) and C6 cells expressing control vector (CV) (Fig. 6). The size of *NHE1*-knockdown tumors (NHE1-KD) was slightly smaller than C6 wt or CV tumors, but significantly larger than that of NHE5-KD tumors. Histological characterization revealed that C6 wt cells and CV cells showed severe invasion into the surrounding tissue whereas invasion of *NHE5*-knockdown cells to the surrounding tissue was not apparent (Fig. 7). The Ki-67 labeling index was significantly lower in NHE5-KD tumors than in CV tumors (Fig. 8).

## Discussion

C6 cells express both NHE5 and NHE1, which makes them a unique model in which to investigate the role of NHE5 and NHE1 in tumor growth, invasion, and signaling. Using C6 glioma cell lines stably expressing plasmid-based shRNA as a model, we demonstrated that *NHE5*-knockdown greatly inhibits tumor growth. Although subcutaneous tissue is different from actual environment in the brain, it is useful to assess the malignant potential, such as proliferation and invasion as an in vivo model. The microscopic appearance of the tumor migration front, the Ki-67 labeling index of tumor xenografts, invasion assays through Matrigel, and spheroid growth experiments all suggest that *NHE5*-knockdown cells are less invasive. *NHE5*-depletion downregulates the signaling pathways mediated by MET, EGFR, and integrins, whereas the influence of *NHE1*-depletion on these signaling pathways was limited. Amplification of EGFR is the most commonly observed genetic abnormality in glioma, and more than 50% of glioma cases are said to be associated with amplification of EGFR [30]. However, EGFR-targeted therapies have been largely ineffective for glioma due to the rapid development of drug resistance [31]. Multiple growth factor receptors including MET and EGFR are known to be overexpressed in aggressive gliomas [31–34]. As different growth factor receptors share common downstream signaling pathways, and inhibition of one growth factor receptor may upregulate other growth factor receptors, blockade of a common upstream regulatory mechanism of growth factor receptors has potential as a potent anti-cancer therapy and may enable resistance against anti-EGFR therapy to be overcome [35, 36]. In this respect, our current findings indicate NHE5 as a potential therapeutic target for glioma.

Defective attachment to collagen is a unique phenotype found in NHE5-depleted glioma cells but not in NHE1-depleted cells. We have shown that NHE5 has a critical role in endocytic recycling and degradation of integrin β1, which likely accounts, at least in part, for the defective adhesion of *NHE5*-knockdown glioma cells. Coordinated recyclings of MET, EGFR, and other RTKs with Integrins through Rab11-FIP1 have been suggested to play a pivotal role in promoting cancer invasion [37, 38]. In addition, EGFR signaling has been implicated in trafficking of Integrin α5β1 to the leading edge of migrating cells [37]. NHE5 potently acidifies the lumen of recycling endosomes and facilitates the return of MET and TRKA to the plasma membrane [14, 16]. However, it is not known whether NHE5 influences intracellular targeting and degradation of membrane receptors other than growth factor receptors. Our current findings suggest that NHE5 impacts on the function of a broader range of membrane receptors including growth factor receptors and integrins by balancing the recycling and degradation of internalized receptors.

Amiloride derivatives such as 5-(*N*-ethyl-*N*-isopropyl) amiloride (EIPA) and 5-(*N*,*N*-hexamethylene)-amiloride (HMA) are known to be NHE1-specific inhibitors with inhibition constants of 15 nM and 13 nM, respectively [39, 40]. These NHE1 inhibitors are also able to inhibit NHE5; however, approximately 30-fold higher doses are required to sufficiently inhibit heterologously expressed NHE5 in rodent fibroblasts [39]. It is possible that an even greater concentration of these inhibitors is required to block endogenous NHE5 activity in recycling endosomes. Thus, high concentrations may cause off-target effects by inhibiting Na^+^-channels and Na^+^/Ca^2+^-ATPases [40]. Stock and colleagues reported that losartan, an angiotensin II type 1 receptor antagonist, inhibits NHE1 activity and migration, but unfortunately increased adhesion and invasion in human melanoma (MV3) cells [8]. On the other hand, a previous screen of chemical derivatives of EIPA on Na^+^/H^+^ exchanger-deficient fibroblasts that heterologously express NHE3 led to the identification of NHE3-specific inhibitors. As NHE3 and NHE5 share similar pharmacological and physiological transporter properties, and the transporter activity of NHE3 and NHE5 can be determined in a similar assay [15], it is possible that an analogous drug screening strategy used to isolate NHE3 inhibitors may identify novel NHE5 inhibitors.

Although NHE1 inhibitors are known to effectively retard cell proliferation of different malignant tumor cells [8–11], we have shown that *NHE5* knockdown by shRNA markedly diminishes the proliferation and invasion of C6 glioma cells, and demonstrate that *NHE5* knockdown has a significantly different effect on tumor growth and invasion in mouse xenografts compared to the impact caused by *NHE1* knockdown. We previously showed that some, but not all, phenotypes of *NHE5*-knockdown cells are rescued by re-expression of shRNA-resistant *NHE5* (functional *NHE5* that has a mismatch sequence against the shRNA target region). In particular, we found that phenotypes related to intracellular targeting of MET and growth factor signaling tend not to be rescued [13, 14]. In this regard, it is interesting to note that re-expression of shRNA-resistant *NHE5* did not rescue the slow tumor growth caused by *NHE5* depletion (data not shown). We postulate that physiological NHE5 expression levels within a narrow range allows the right balance between endocytic recycling and degradation of cell surface receptors including EGFR, MET, and integrins, thereby tightly regulating any signaling through these receptors.

## Conclusion

Our current study presents a new hypothesis wherein neuron-type NHE5 ectopically expressed in C6 glioma cells plays a critical role in MET, EGFR, and integrin signaling in glioma. If NHE5 expression is restricted to glioma lesions, NHE5 may add diagnostic and therapeutic value. In future studies, it is important to investigate whether NHE5 expression is upregulated in pathological samples obtained from glioma patients, and identify whether there are any correlations between NHE5 expression and pathological type, prognosis, and other glioma-associated factors.

## Abbreviations

NHE: Na^+^/H ^+ ^exchanger
FBS: Fetal bovine serum
ECM: Extra cellular matrix
EGFR: Epidermal growth factor receptor
HGF: Hepatocyte growth factor
hITGB1: Human integrin β1
RIPA: Radioimmunoprecipitation assay
SDS: Sodium dodecyl sulfate
PAGE: Poly-acrylamide gel electrophoresis
APH: Acid phosphate assay
PFA: Paraformaldehyde
PEI: Polyethyleneimine
PBS: Phosphate buffered saline
HA: Human influenza hemagglutinin
DMEM: Dulbecco’s modified Eagle’s medium
NKA: Sodium/potassium-ATPase
TRKA: Tropomyosin receptor kinase A
